# Synthesis of self-assembled nucleobases and their anhydrous proton conductivity†

**DOI:** 10.1039/c9ra06841d

**Published:** 2019-11-08

**Authors:** Masanori Yamada, Kento Tanoue

**Affiliations:** Department of Chemistry, Faculty of Science, Okayama University of Science Ridaicho, Kita-ku Okayama 700-0005 Japan myamada@chem.ous.ac.jp +81 86 256 9757 +81 86 256 9550

## Abstract

We synthesized self-assembled nucleobases (SANs), such as 1-dodecylthymine (DOT) or 9-dodecyladenine (DOA), in which the nucleobase is immobilized on a long alkyl chain. The thermal stability of the SAN was increased by mixing with the acidic surfactant mono-dodecyl phosphate (MDP). Additionally, the SAN–MDP composite material showed proton conductivity of 4.62 × 10^−4^ S cm^−1^ at 160 °C under anhydrous conditions. Additionally, the activation energy of the proton conduction was approximately 0.2 eV and this value was one order of magnitude higher than that of a typical humidified perfluorinated membrane, in which the proton can be moved by vehicle molecules, such as water molecules. In contrast, when the nucleobase without the immobilization of a long alkyl chain was mixed with MDP, the proton conductivity of these composite materials was two orders of magnitude less than that of the SAN–MDP composite. Therefore, we measured the XRD spectra of the SAN–MDP composite material. As a result, the SAN–MDP composite material showed a self-assembled structure with a two-dimensional proton conducting pathway, such as a lamellar structure, and that the anhydrous proton conduction was related to the interaction between the nucleobase of the SAN and the phosphate group of MDP. Consequently, the self-assembled nucleobase derivatives have the potential for use as novel anhydrous proton conductors with a two-dimensional proton conducting pathway.

## Abstract 2

We synthesized the proton conductive self-assembled nucleobase, such as 1-dodecylthymine and 9-dodecyladenine, in which the nucleobase is immobilized on a long alkyl chain.

## Graphical abstract

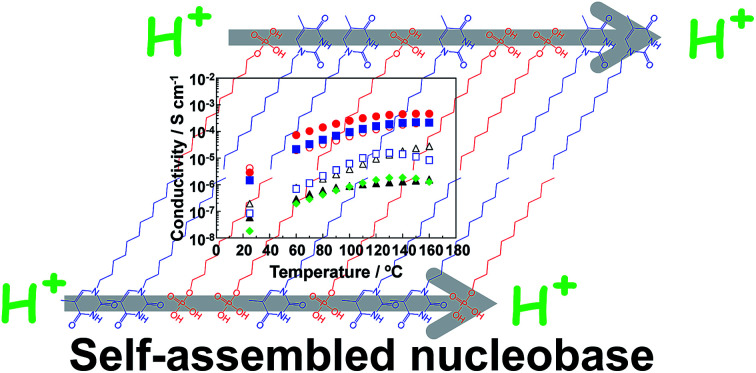

## 1. Introduction

The anhydrous proton conductor is an important material in a polymer electrolyte membrane fuel cell (PEFC) which is operated at intermediate temperatures (100–200 °C) or under anhydrous conditions.^1–5^ Generally, the proton conducting mechanism can be divided into the vehicular mechanism and the non-vehicular mechanism.^6–8^ In the vehicular mechanism, the proton in the electrolyte transfers, with diffusible vehicle molecules such as the oxonium ions. In this case, water molecules are essential for the proton transfer and the proton conduction does not occur under low humidity and anhydrous conditions. On the other hand, in the non-vehicular mechanism, the proton in the electrolyte transfers from the protonated site to the non-protonated site without diffusible water molecules by thermal energy. Therefore, the proton conduction can occur under low humidity and anhydrous conditions.^1–5^ In particular, the non-vehicular mechanism has been reported for a composite material consisting of acidic molecules and basic heterocyclic molecules, such as imidazole and benzimidazole.^9,10^ In the acid–base composite materials, the basic heterocyclic molecules accepted the free proton from the acidic molecule and formed an acid–base composite through an electrostatic interaction. As a result, the proton transfer occurred from the protonated heterocyclic molecule to the non-protonated heterocyclic molecule.^4–8^ In this case, the protonated and non-protonated heterocyclic molecules can play the role as a proton donor and acceptor, respectively.

For anhydrous proton conduction, the molecular structure of the acid–base composite material is crucial for the rate of proton transport.^7,10,11^ Namely, the existence of a proton conducting pathway in a material is one of the most important factors for the high proton conductor.^11–13^ However, the simple mixing of acidic and basic molecules generally forms a random proton conducting pathway and, as a result, does not provide the maximum proton conductivity. Therefore, various anhydrous proton conductors with the proton conducting pathway, such as periodic-mesoporous-organosilica encapsulated imidazole molecule,^14^ self-assembled organic phosphonates consist of long alkyl chains,^15^ and Kevlar® nanofibers with cadmium telluride nanocrystals and phosphoric acid,^16^ have been reported and these materials showed the anhydrous proton conductivity of 7.11 × 10^−3^ S cm^−1^ at 180 °C, 10^−2^ S cm^−1^ at 140 °C, and 2.35 × 10^−1^ S cm^−1^ at 160 °C, respectively. We previously prepared the self-assembled acid–base composite material by mixing the acidic surfactant, mono-dodecyl phosphate (MDP), and the basic surfactant, undecyl imidazole (UI), and estimated its anhydrous proton conduction.^11^ Consequently, the MDP-UI composite material formed a highly-ordered lamellar structure with a two-dimensional proton conducting pathway and showed a high proton conductivity under anhydrous conditions.

DNA, the most important genetic material of living organisms, is a natural product and safe for humans and the environment.^17–20^ Additionally, DNA contains various nucleobases, such as adenine (A), guanine (G), thymine (T), and cytosine (C). These nucleobases have the property of a heterocyclic molecule and play the role as a proton conductor. In fact, we previously reported the anhydrous proton conduction of a nucleobase.^19,22^ However, the nucleobase alone could not form a sufficient proton conducting pathway. Therefore, in this strategy, we attempted to synthesize the nucleobase with a long alkyl chain, such as an alkylated-thymine or -adenine, in order to form a proton conducting pathway using its self-assembled property.

**Scheme 1:**
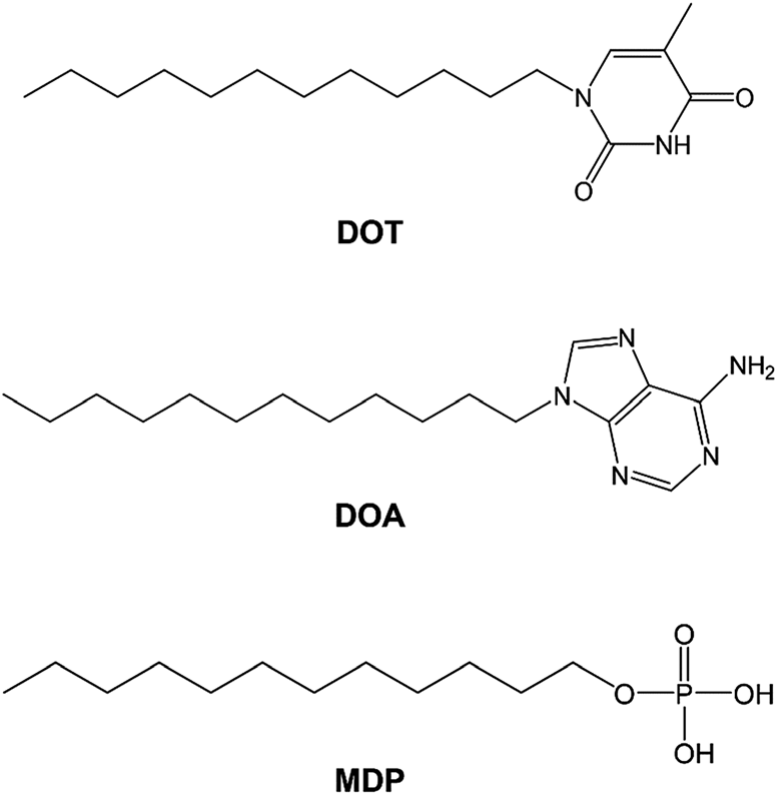
Molecular structure of DOT, DOA, and MDP.

In this study, we synthesized the self-assembled nucleobase (SAN), such as 1-dodecylthymine (DOT) or 9-dodecyladenine (DOA) in which nucleobase is immobilized on a long alkyl chain and prepared the SAN–mono-dodecyl phosphate (MDP) composite material by mixing SAN and MDP. Scheme 1 shows the molecular structures of DOT, DOA, and MDP. The SAN–MDP composite material formed the self-assembled structure with the two-dimensional proton conducting pathway by the interaction between the nucleobase in SAN and the phosphate group in MDP. Consequently, the SAN–MDP composite material indicated the proton conductivity of 4.62 × 10^−4^ S cm^−1^ at 160 °C under anhydrous conditions. In contrast, when the nucleobase without the immobilization of a long alkyl chain was mixed with MDP, the proton conductivity of these composite materials was two orders magnitude less than that of the SAN–MDP composite. Therefore, the SAN derivatives have the potential to be used for novel anhydrous proton conductors with the two-dimensional proton conducting pathway.

## 2. Experimental sections

### 2.1. Material

Thymine (T), adenine (A), 1,1,1,3,3,3-hexamethyldisilazane (HMDS), dodecyl bromide, mono-dodecyl phosphate (MDP), and potassium carbonate were purchased from Tokyo Kasei Industries, Ltd., Tokyo, Japan and Wako Pure Chemical Industries, Ltd., Osaka, Japan. The glass GC-50 filter (thickness: 0.2 mm) was purchased from Advantec Toyo Kaisha, Ltd., Tokyo, Japan. The solvents used were analytical grade in all the experiments.

### 2.2. Synthesis of DOT

**Scheme 2:**
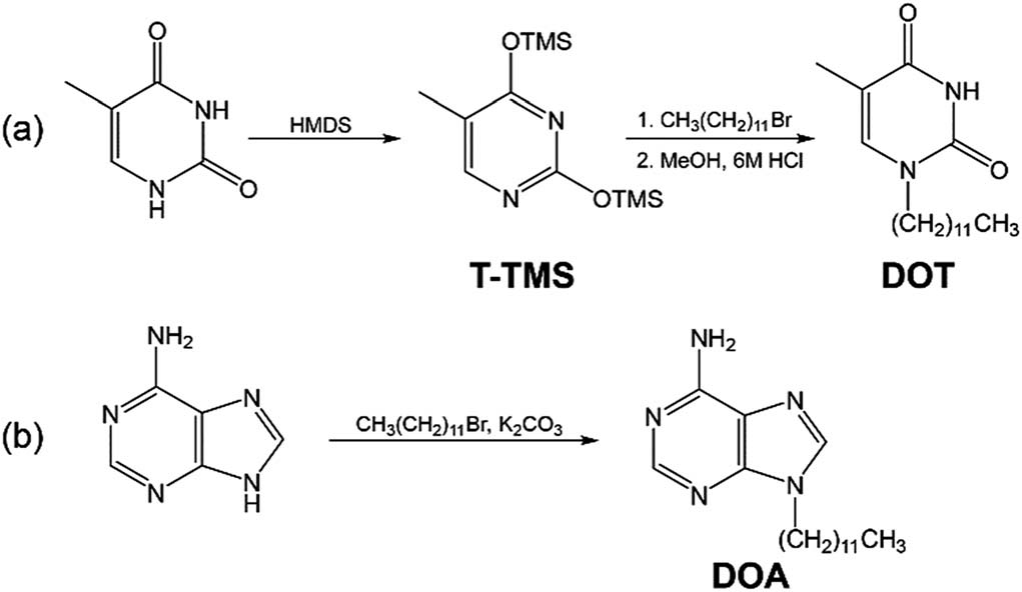
Synthetic scheme of DOT and DOA.

The 1-dodecylthymine (DOT) was synthesized by a partially modified procedure.^23,24^Scheme 2(a) shows the synthetic scheme of the DOT. The thymine (5 g, 39 mmol) and HMDS (57 ml, 296 mmol) were dissolved in DMF (10 ml) and refluxed at 160 °C for 24 hours. After the reaction, DMF and HMDS were removed at 40 °C in a the vacuum oven and the 5-methyl-2,4-bis(trimethylsiloxy)-pyrimidine (T-TMS) was obtained. A mixture of T-TMS (7 g, 26 mmol) and dodecyl bromide (15 g, 63 mmol) was stirred at 60 °C for 10 days and an oily substance obtained. The substance was added to the mixed solution of methanol (200 ml) and 6 M HCl (20 ml), then heated. The mixture was filtered to remove the precipitate. After removing the solvents by a rotary evaporator, the residue was extracted with water/chloroform, then the chloroform was evaporated. The residue was washed with diethyl ether and recrystallized from benzene. The DOT was obtained as a white powder (0.60 g, 7.8%). The identification of the synthetic DOT was demonstrated by its ^1^H NMR spectrum using a JNM-ECS400 (JEOL Ltd., Tokyo, Japan). ^1^H NMR (400 MHz, DMSO-d_6_): *δ* = 11.19 (1H, s, N–H), 7.52 (1H, s, C6–H), 3.59 (2H, t, N–CH2), 1.74 (3H, s, C5–CH3), 1.54 (2H, m, N–C–CH2), 1.27 (18H, m, –CH2–), 0.85 (3H, t, C–CH3). Additionally, the identification of the synthetic DOT was also determined by matrix assisted laser desorption time-of-flight mass spectrometry (MALDI-TOF-MS) (Autoflex speed, Bruker Corporation, Billerica, MA). *m*/*z* 295.358 [M + H]^+^; calcd for C_17_H_30_N_2_O_2_*m*/*z* 294.23.

### 2.3. Synthesis of DOA

The 9-dodecyladenine (DOA) was synthesized by a partially modified procedure.^23^Scheme 2(b) shows the synthetic scheme of the DOA. The adenine (1.35 g, 10 mmol), dodecyl bromide (2.66 g, 10 mmol), and potassium carbonate (1.52 g, 10 mmol) were added to DMF and refluxed at 130 °C for 18 hours with stirring. After the reaction, the mixture was filtered to remove the precipitate and the solvent was removed by a rotary evaporator. The residue was recrystallized with benzene. The DOA was obtained as a white powder (1.9 g, 63%). The identification of DOA was determined by its ^1^H NMR spectrum and MALDI-TOF-MS. ^1^H NMR (400 MHz, DMSO-d_6_): *δ* = 8.10 (2H, s, C2–H, C8–H), 7.14 (2H, s, N–H2), 4.09 (2H, t, N–CH2), 1.76 (2H, m, N–C–CH2), 1.18 (18H, m, –CH2–), 0.82 (3H, t, C–CH3). *m*/*z* 304.508 [M + H]^+^; calcd for C_17_H_29_N_5_*m*/*z* 303.24.

### 2.4. Preparation and characterization of SAN–MDP composite material

The SAN–MDP composite material was prepared as follows: the SAN and MDP were dissolved in ethanol or chloroform. The SAN and MDP solutions were simultaneously cast on a Teflon® plate and dried at room temperature overnight. The proton conducting samples were prepared as follows: the SAN and MDP solutions were simultaneously cast on a glass filter (7 × 7 mm^2^) and dried at room temperature overnight. The molar ratio (mol%) of the MDP in the SAN–MDP composite material was determined by [mol% of MDP in SAN–MDP composite material] = ([mole of MDP]/([mole of SAN] + [mole of MDP])) × 100. The molar ratios of the MDP were 0–100 mol%. The thymine–MDP and adenine–MDP composite materials were obtained by a similar procedure.

The DOT–DOA–MDP composite material was prepared as follows: the mixed DOT–DOA solution was prepared by dissolving the DOT and DOA (1 : 1 molar ratio) in chloroform. The mixed DOT–DOA and MDP solutions were simultaneously cast on a glass filter and dried at room temperature overnight.

The thermal stability of the SAN–MDP composite material was analyzed by a thermogravimetric-differential thermal analysis (TG-DTA) (DTG-60, Shimadzu Corp., Kyoto, Japan). The TG-DTA measurement of the composite materials was carried out at the heating rate of 10 °C min^−1^ under flowing dry-nitrogen. The sample weight for the TG-DTA measurements was normalized at 1 mg. The IR spectrum of the composite material was measured using an FT-IR 8400 Fourier transform infrared spectrometer (Shimadzu Corp.) with the diamond attenuated total reflection (ATR) prism. The resolution of the spectrum was 4 cm^−1^. The X-ray diffraction (XRD) powder patterns of the composite materials were measured by a Philips X'Pert (Royal Philips, Amsterdam, Netherlands) using CuK_α_ radiation (*λ* = 1.54 Å).

### 2.5. Anhydrous proton conductive measurement

The proton conductivity of the SAN–MDP composite material was demonstrated by the a.c. impedance method in the frequency range from 4 Hz to 1 MHz using a chemical impedance analyzer 3532-80 (Hioki Co., Nagano, Japan) in a stainless steel vessel from room temperature to 160 °C under flowing dry-nitrogen. The SAN–MDP composite material was sandwiched between two gold electrodes (*ϕ* 5 mm).^11,21^ The direction of conductive measurement was perpendicular to the composite material. The conductivities were determined from a typical impedance response (Cole–Cole plots). The resistances of the composite materials were obtained from extrapolation to the real axis.

Before the measurements of the proton conductions, the composite materials were heated at 160 °C in a stainless steel vessel for 3 hours to evaporate the water and volatile components from the proton conductive samples. Furthermore, all the experimental procedures for the proton conduction were carried out under flowing dry-nitrogen. Therefore, the measured impedance response indicated the anhydrous proton conduction of the materials.^4,11,21^

## 3. Results and discussion

### 3.1. Synthesis of DOT and DOA

The DOT and DOA were synthesized by the reaction of the nucleobase and dodecyl bromide. The identification was obtained by ^1^H NMR and MS spectrometry. After the reaction, the signal of the imino proton in the nucleobase, attributed to the 1 position of the pyrimidine ring and 9 position of the purine ring, disappeared. Additionally, the synthesized DOT and DOA showed signals of *δ* = *ca.* 1.2 and *ca.* 0.8, related to the –CH_2_– and terminal –CH_3_ in the long alkyl chain, respectively. These results indicated alkylation at the 1 position of the pyrimidine ring or the 9 position of the purine ring. Furthermore, the MALDI-TOF-MS showed molecular ion peaks at 295.358 and 304.508, related to the DOT and DOA, respectively.

We next estimated the dissolution of the SAN into the solvent. Although the synthesized SAN, such as DOT and DOA, did not show any dissolution in water, these compounds could dissolve in an organic solvent, such as ethanol or chloroform. Therefore, the SAN and MDP were dissolved in ethanol or chloroform. The SAN–MDP composite materials were prepared by the casting of each solution onto Teflon® plates. The obtained white powder was analyzed by XRD, TG-DTA, and IR measurements. On the other hand, the powdered SAN–MDP composite material could not be prepared as a pellet by pressure formation using a press after a long time. Therefore, the proton conducting samples were prepared as follows: each solution was simultaneously cast onto a glass filter and dried. The total amount of the SAN and MDP in the proton conducting sample was 10 μmol.

### 3.2. Thermal stability of SAN–MDP composite material

**Fig. 1:**
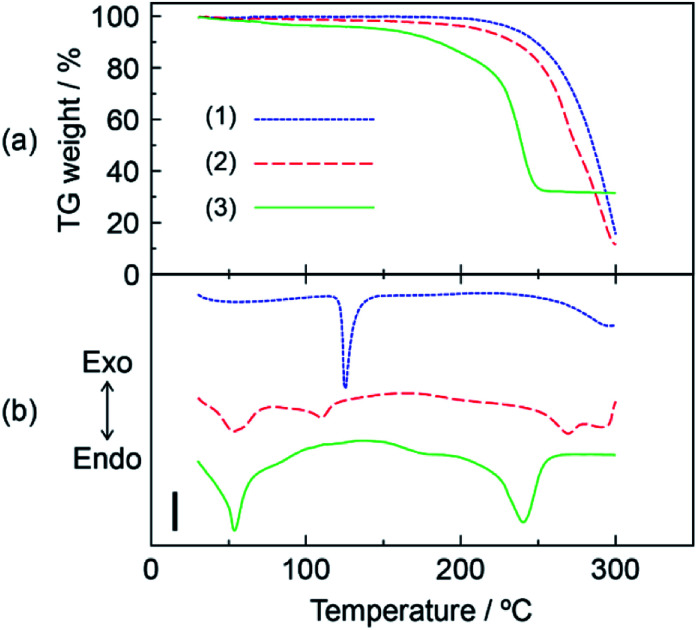
TG (a) and DTA (b) curves of (1) DOT material, (2) DOT–40 mol% MDP composite material, and (3) MDP material. These measurements were done at the heating rate of 10 °C min^−1^ under flowing dry-nitrogen. The TG-DTA measurements were normalized at 1 mg. Scale bar in (b) indicates 10 μV mg^−1^. Triplicate experiments gave similar results.

The thermal stability of the DOT–MDP composite material was analyzed by thermogravimetric (TG) and differential thermal analyses (DTA). Fig. 1(a) and (b) show the TG and DTA of (1) DOT without the mixing of MDP, (2) DOT–40 mol% MDP composite material, and (3) MDP without the mixing of DOT. These measurements were done under a flowing dry-nitrogen. The DOT without the mixing of MDP indicated an endothermic peak related to the melting of DOT at 121.4 °C. In addition, at >200 °C, the DOT showed a high TG weight loss due to evaporation of the DOT. The MDP without the mixing of the DOT indicated two endothermic peaks at 48.0 °C and 224.1 °C. Each endothermic peak has been attributed to the melting and boiling of MDP.^21^ In contrast, the DOT–MDP composite materials did not show the endothermic peak related to the melting of the DOT and MDP. Additionally, the TG weight loss of the composite material was a few percent at or below 200 °C due to the evaporation of water and dehydration of the phosphate group in the MDP. This thermal stabilization of the DOT–MDP composite material is due to the interaction between the DOT and MDP molecules. Similar results, such as the thermal stabilization by the addition of MDP, have been reported for various acid–base composite materials.^11,21^ These results suggested that the DOT–MDP composite material does not produce a diffusible ion by the melting and structural change of the samples and is stable at an intermediate temperature (<160 °C). Therefore, in further experiments, the SAN–MDP composite material were heated at 160 °C for 3 hours to evaporate the water and volatile components from the composite material. The heat-treated sample was immediately used for the next measurement.

### 3.3. Molecular structure and property of SAN–MDP composite material

**Fig. 2:**
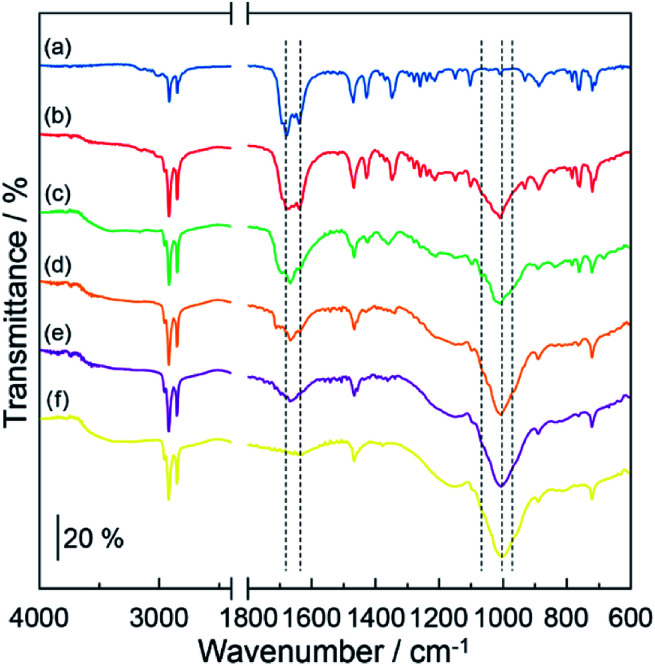
IR spectra of DOT–MDP composite materials. (a), DOT material; (b), DOT–20 mol% MDP composite material; (c), DOT–40 mol% MDP composite material; (d), DOT–60 mol% MDP composite material; (e), DOT–80 mol% MDP composite material; and (f), MDP material. These samples were prepared by heating at 160 °C for 3 hours. The IR spectrum was measured at the resolution of 4 cm^−1^. Triplicate experiments gave similar results.

Fig. 2 shows the IR spectra of (a) DOT without the mixing of MDP, (b) DOT–20 mol% MDP composite material, (c) DOT–40 mol% MDP composite material, (d) DOT–60 mol% MDP composite material, (e) DOT–80 mol% MDP composite material, and (f) MDP without mixing of the DOT. The DOT without mixing of the MDP showed an absorption band at 1631 cm^−1^ and 1681 cm^−1^, attributed to the lactam form of thymine.^21,26,27^ When the MDP molecules were added to the DOT, the absorption band at 1660 cm^−1^, related to the lactim form of thymine,^21,25–27^ increased. These results suggested that the molecular structure of thymine in the DOT partially changes from the lactam form to the lactim form (lactam–lactim tautomerism) by mixing of the MDP molecules. Similar phenomena have been reported for the acid-uracil composite material.^21^

On the other hand, the MDP molecules with mixing of the DOT showed two new shoulder bands at 1066 cm^−1^ and 960 cm^−1^, the stretching vibration of –HPO_4_^−^ and –PO_4_^2−^,^21,26,28,29^ respectively (see spectrum (c) in Fig. 1). Additionally, the absorption band at *ca.* 1000 cm^−1^, attributed to the asymmetric stretching vibration of the P–OH group of MDP,^21,26^ relatively decreased by mixing of the DOT (see spectra (f) and (c) in Fig. 2). This is due to the deprotonation of the P–OH group of MDP by the addition of DOT. In fact, the shoulder bands, which are related to the ionization of the P–OH group appeared at 1066 cm^−1^ and 960 cm^−1^ in the composite materials. Similar phenomena, such as the decrease or disappearance of an absorption band by the deprotonation, have been reported for various acid–base composite materials.^4,11,21^ These results suggested that the DOT and MDP form an acid–base composite structure, and that the thymine portion in the DOT converts the lactam form to the lactim form by the lactam–lactim tautomerism.

### 3.4. XRD measurements of DOT–MDP composite material

**Fig. 3:**
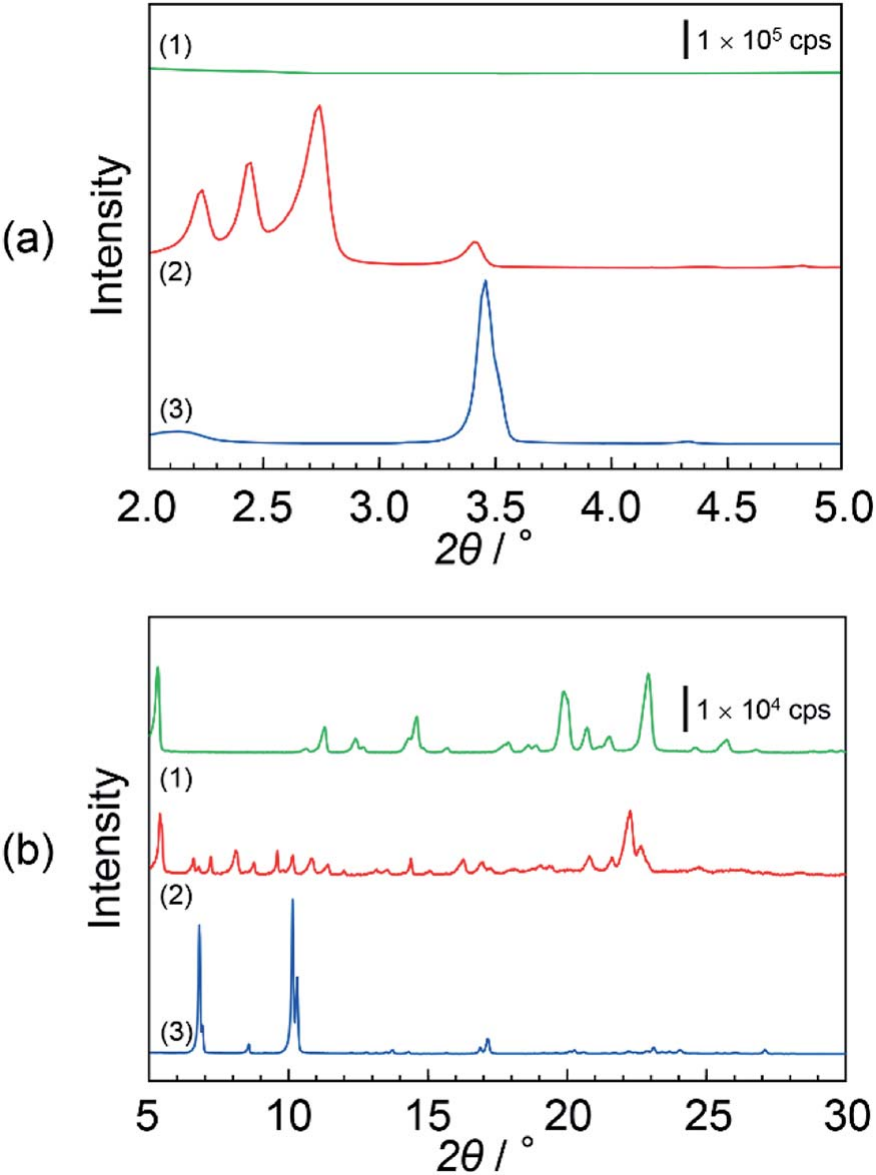
XRD patterns of (1) DOT material, (2) DOT–40 mol% MDP composite material, and (3) MDP material. (a) and (b) show the XRD patterns in the low- and high-angle regions, respectively. These samples were prepared by heating at 160 °C for 3 hours. Triplicate experiments gave similar results.

Fig. 3(a) and (b) show the XRD pattern of (1) DOT material, (2) DOT–40 mol% MDP composite material, and (3) MDP material in the low- and high-angle regions, respectively. The DOT material without the mixing of MDP showed diffraction peaks at 5.32°, 11.26°, 14.57°, 19.85°, and 22.90°. Additionally, the MDP material without the mixing of DOT indicated diffraction peaks at 3.45°, 6.91°, and 10.14°. Therefore, we demonstrated the XRD measurements of the DOT–40 mol% MDP composite material. Surprisingly, for the DOT–40 mol% MDP composite material, new diffraction peaks at 2.23°, 2.44°, 2.74°, and 22.3° appeared. Additionally, the diffraction peak at 3.45°, 6.91°, and 10.14°, related to the MDP, significantly decreased or disappeared. According to Bragg's equation, the *d*-spacing at 2.23°, 2.44°, 2.74°, and 22.3° were calculated to be 39.6 Å, 36.2 Å, 32.3 Å, and 3.99 Å, respectively. Since the diffraction peaks at 2.74° (32.3 Å) indicated the highest intensity, the *q* space (2π/*d*-spacing) was calculated from the *d*-spacing. Fig S1 and Table S1 in the ESI† show the diffraction patterns with *q* space as the *x*-axis and the value of *q* space, respectively. As a result, in *q* space, the diffraction peak at 2.74° of DOT–40 mol% MDP composite material indicated a secondary peak related to the two-dimensional lamellar structure (see Fig. S1 and Table S1 in ESI†). Additionally, the *d*-spacing of 3.99 Å was the same as the distance between the alkyl chains in the close-packed structure, and similar results have been reported for the self-assembled acid–base composite materials.^11^ Furthermore, the molecular sizes of DOT and MDP, which were estimated from the molecular modelling, were 20.3 Å and 19.1 Å, respectively, and this sum was approximately 40 Å. Therefore, the DOT–MDP composite material is not vertically oriented but arranged with the distortion of *ca.* 54°.

**Scheme 3:**
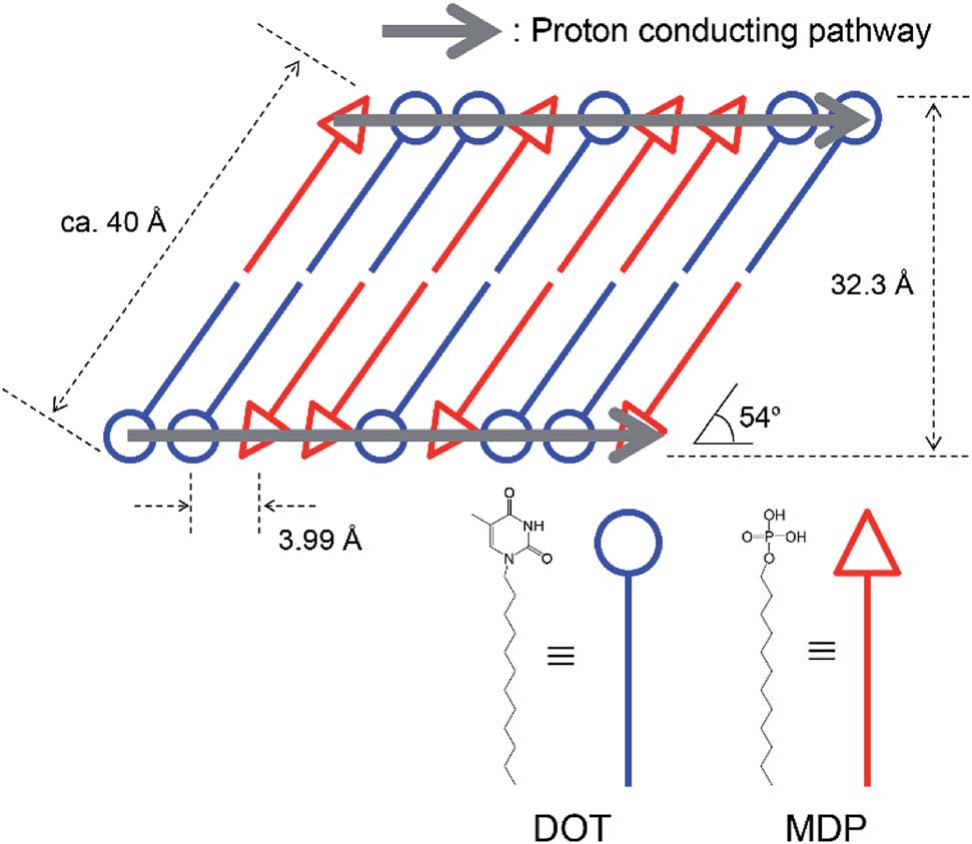
Structural model with the proton conducting pathway of the DOT–MDP composite material. The distances between the molecules were estimated from the XRD measurements and the molecular modelling.

Scheme 3 shows the structural model of the DOT–40 mol% MDP composite material. The DOT and MDP molecules formed a self-assembled structure, such as lamellar structure, with an interaction between the long alkyl chains. Additionally, the long alkyl chains of DOT and MDP were arranged with the distortion of *ca.* 54°. Furthermore, the thymine in the DOT and the phosphate group in the MDP are closely aligned by the acid–base interaction in the composite material. As a result, the head group of DOT and MDP may have the potential for a two-dimensional proton conducting pathway without the assistant of a vehicular molecule.

### 3.5. Anhydrous proton conduction of DOT–MDP composite material

The proton conduction of the DOT–MDP composite material with a two-dimensional proton conducting pathway under anhydrous conditions was measured by an impedance analyzer. The obtained impedance responses (Cole–Cole plots) of the DOT–MDP composite material were similar to that of the acid–base composite material with the highly-anhydrous proton conductivity.^4,11,21^ We estimated the proton conductivity under anhydrous conditions from the resistances of the composite materials.

**Fig. 4:**
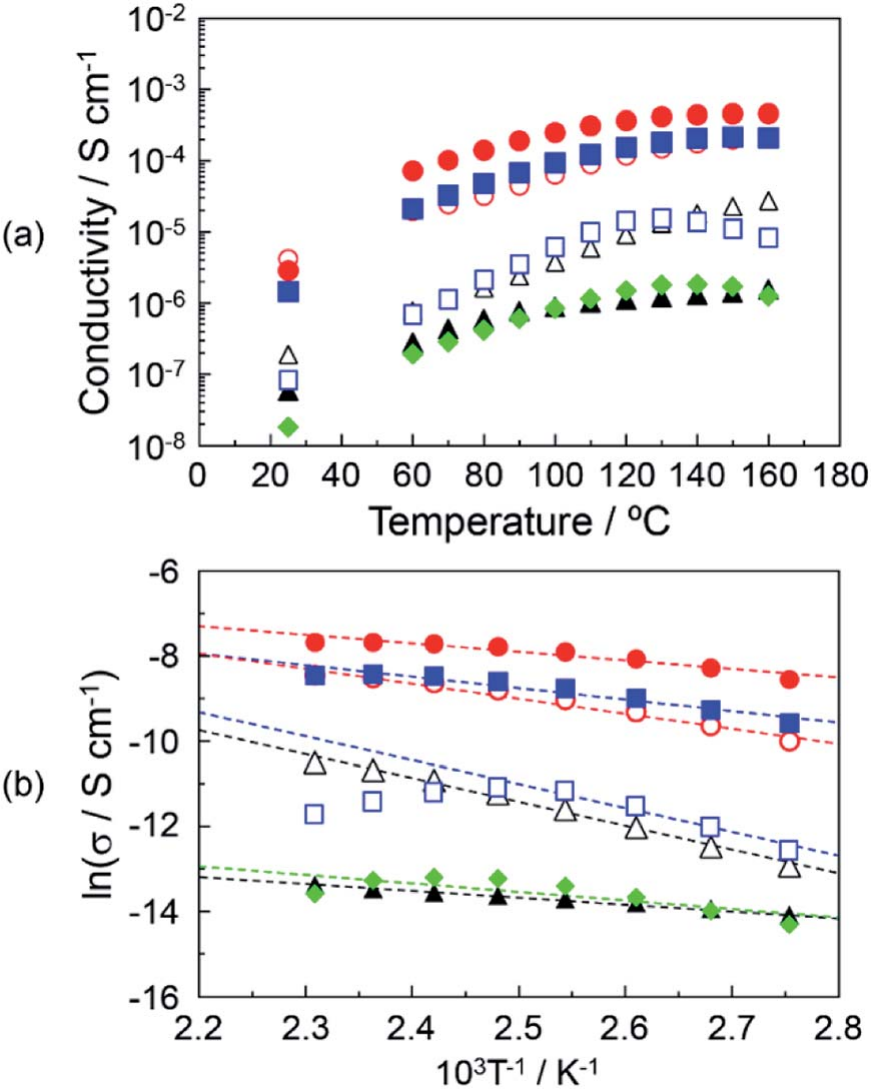
(a) Proton conductivities of DOT–MDP composite materials. (b) Arrhenius plots of the proton conductivity of the DOT–MDP composite materials. The solid lines of the DOT and DOT–MDP composite materials are the results of a least-squares fitting. The least-square fitting of MDP was calculated in the range of 90–130 °C. (▲), DOT material; (△), DOT–25 mol% MDP composite material; (●), DOT–40 mol% MDP composite material; (○), DOT–60 mol% MDP composite material; (■), DOT–80 mol% MDP composite material; (□), MDP material; and (♦), thymine–40 mol% MDP composite material. The proton conductive measurements were demonstrated under anhydrous conditions. Triplicate experiments gave similar results.

Fig. 4(a) shows the proton conductivity under the anhydrous conditions of (▲), DOT without the mixing of MDP; (△), DOT–25 mol% MDP composite material; (●), DOT–40 mol% MDP composite material; (○), DOT–60 mol% MDP composite material; (■), DOT–80 mol% MDP composite material; (□), MDP without the mixing of DOT; and (♦), thymine–40 mol% MDP composite material. The proton conduction increased with the temperature. The maximum proton conductivities of the DOT and MDP without the mixing were 1.6 × 10^−6^ S cm^−1^ at 160 °C and 1.6 × 10^−5^ S cm^−1^ at 130 °C, respectively. Therefore, the proton conductive measurements of the DOT–MDP composite material, which formed the self-assembled structure, were demonstrated. The proton conductivity of the DOT–MDP composite material increased with the addition of MDP and reached a maximum value of 4.6 × 10^−4^ S cm^−1^ at 40 mol% MDP. To estimate the effect of the two-dimensional proton conducting pathway, the proton conductive measurements of the thymine–40 mol% MDP composite material, which cannot form a proton conducting pathway, were demonstrated. As a result, the proton conduction of the thymine–40 mol% MDP composite material was 1.26 × 10^−6^ S cm^−1^ at 160 °C and this value was two orders magnitude less than that of the DOT–MDP composite material (see ♦ in Fig. 4(a)). In addition, the proton conductivity of MDP and thymine–40 mol% MDP composite material decreased at 130 °C. This is due to the dehydration of phosphate group in MDP, which could not form a composite structure. These results suggested that the self-assembled structure of the DOT–MDP composite material plays an effective role in the two-dimensional proton conducting pathway.

Fig. 4(b) shows the Arrhenius plot of the proton conductivity of (▲), DOT without the mixing of MDP; (△), DOT–25 mol% MDP composite material; (●), DOT–40 mol% MDP composite material; (○), DOT–60 mol% MDP composite material; (■), DOT–80 mol% MDP composite material; (□), MDP without the mixing of DOT; and (♦), thymine–40 mol% MDP composite material. The solid lines for the DOT and composite material indicated the results of a least-squares fitting. The least-squares fitting of MDP was calculated in the range of 90–130 °C. Since the proton conduction of the DOT–MDP composite material could be plotted as a straight line from 90–160 °C, we estimated the activation energy (*E*_a_) of the proton conduction from the slope of the Arrhenius plot. The estimated *E*_a_ values were 0.13–0.48 eV. These values were one order higher than that of the humidified proton conductor, such as phosphotungstic acid and typical humidified perfluorinated membrane, in which the proton conduction was based on the diffusible vehicle molecules in the materials.^30–32^ In addition, these *E*_a_ values of the DOT–MDP composite were the same as those of various anhydrous proton conductors.^11,21^ These results suggested that the proton conduction was based on a mechanism without the assistant of water molecules.

On the other hand, the proton conductivities of DOT–MDP composite material without the heat treatment at 160 °C for 3 hours were higher than anhydrous proton conductivity (data not shown). This is because the SAN–MDP composite materials without the heat treatment contained the water contents in its and showed the proton conduction with the assistance of diffusible vehicle molecules, such as oxonium ion. Similar phenomenon, such as the increase of proton conductivity under humidified conditions, has been reported for other proton conductor.^33^

### 3.6. Anhydrous proton conduction of DOT–DOA–MDP composite material

To estimate the contribution of the thymine's proton in DOT to the anhydrous proton conductive mechanism, the proton conductive measurements of the DOT–DOA composite material were demonstrated. Generally, the thymine and adenine molecules form a base pairing by hydrogen bonding.^17,34^ If the thymine's proton in DOT and the adenine's nitrogen in DOA interact by hydrogen bonding, the conductive proton, which is related to the proton conduction in the DOT–DOA composite material, cannot move to a neighbouring site by the formation of base pairing. As a result, the proton conductivity of the DOT–DOA composite material is expected to decrease. The DOT–DOA composite material was prepared by mixing DOT and DOA in the molar ratio of 1 : 1.

**Fig. 5:**
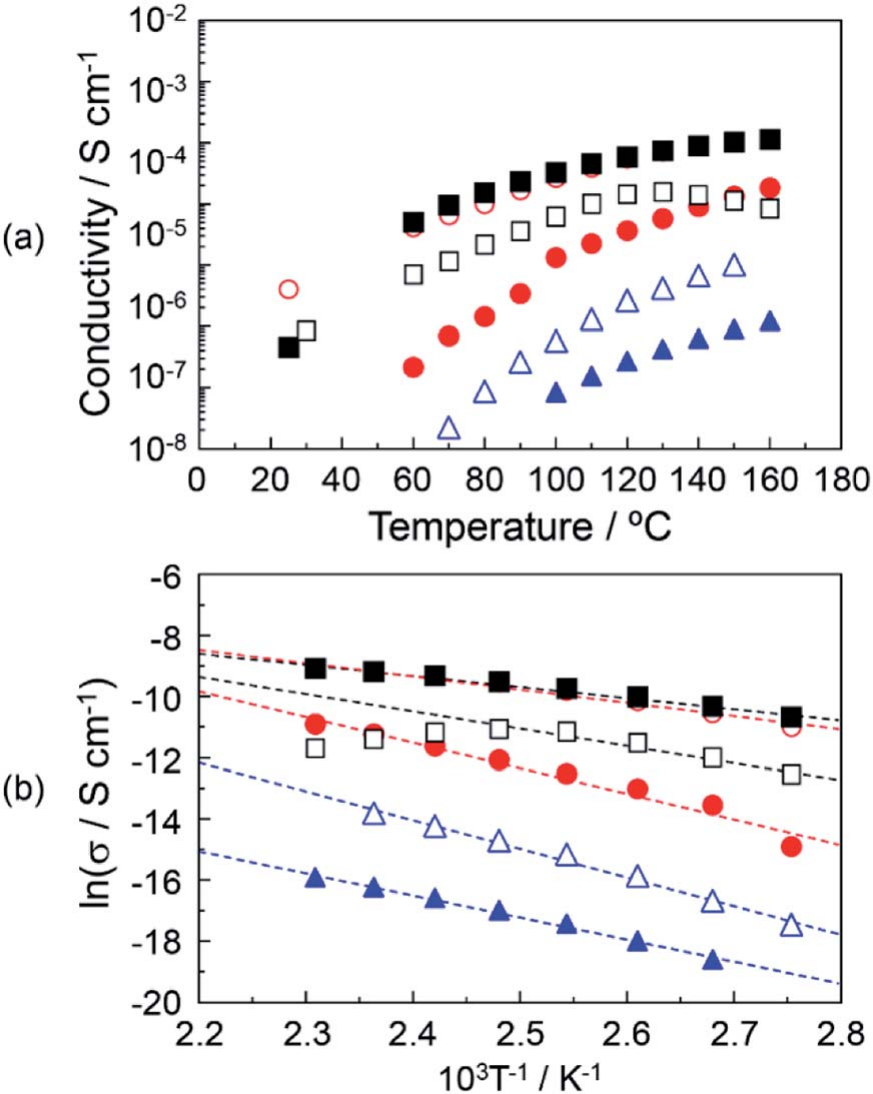
(a) Proton conductivities of DOT–DOA–MDP composite materials. (b) Arrhenius plots of the proton conductivity of the DOT–DOA–MDP composite materials. The solid lines of the DOT–DOA–MDP composite materials are the results of a least-squares fitting. The least-square fitting of MDP was calculated in the range of 90–130 °C. (▲), DOT–DOA material; (△), DOT–DOA–20 mol% MDP composite material; (●), DOT–DOA–40 mol% MDP composite material; (○), DOT–DOA–60 mol% MDP composite material; (■), DOT–DOA–80 mol% MDP composite material; and (□), MDP material. The DOT–DOA material was prepared by mixing DOT and DOA in the molar ratio of 1 : 1. The proton conductive measurements were done under anhydrous conditions. Triplicate experiments gave similar results.

Fig. 5(a) shows the proton conductivity under the anhydrous conditions of (▲), DOT–DOA material without the mixing of MDP; (△), DOT–DOA–20 mol% MDP composite material; (●), DOT–DOA–40 mol% MDP composite material; (○), DOT–DOA–60 mol% MDP composite material; (■), DOT–DOA–80 mol% MDP composite material; and (□), MDP without the mixing of DOT–DOA. The maximum proton conductivity at 160 °C was 1.13 × 10^−4^ S cm^−1^ for the DOT–DOA–80 mol% MDP composite material, and this value was less than a quarter of the maximum proton conductivity for DOT–40 mol% MDP (see ● in Fig. 4(a) and ■ in Fig. 5(a)). Fig. 5(b) shows the Arrhenius plot of the proton conductivity of the DOT–DOA–MDP composite material. The *E*_a_ of the proton conduction was 0.17–0.62 eV. These values were also one order higher than that of the humidified proton conductor.^30–32^

On the other hand, the proton conduction of the DOA–MDP composite material without the mixing of DOT was a low value and the maximum proton conduction at 160 °C was 6.72 × 10^−7^ S cm^−1^ for the DOA–80 mol% MDP composite material under anhydrous conditions (data not shown). This conductive value was three orders of magnitude lower than that of the DOT–40 mol% MDP composite material. These results suggested that the anhydrous proton conduction on the order of 10^−4^ S cm^−1^ was based on the thymine's proton in the DOT molecule rather than the proton of the phosphate group in the MDP molecule and the proton conducting mechanism was related to the lactam–lactim tautomerism.

### 3.7. Anhydrous proton conduction through the two-dimensional proton conducting pathway

We previously reported the anhydrous proton conductor of the acid-heterocyclic molecule composite material at an intermediate temperature.^11,21^ In these cases, the proton conductive mechanism has been based on the Grotthuss-type mechanism, in which the proton transport in the composite material can occur from protonated molecules, such as a protonated heterocyclic molecule, to the non-protonated neighbour molecules.^4–8,11,21^ As a result, the protonated and non-protonated heterocyclic molecules behaved as proton donors and acceptors in the composite material, respectively. Additionally, the acid molecules, such as the phosphate and sulfonic acid groups, acted as proton sources for the heterocyclic molecule.^1,10,11,21,22,35,36^ Therefore, the distance between the proton conductive molecules is one of the most important factors for anhydrous proton conduction. In our research, the SAN and MDP were closely packed by the self-assembled function and the distance between the protonated and non-protonated molecules was *ca.* 4 Å (see Scheme 3). The transport of the proton in the SAN–MDP composite material can occur through the lactam–lactim tautomerism. A similar proton conduction, such as the proton conduction through the lactam–lactim tautomerism, was reported for the acid-uracil composite material.^21,22^ Therefore, the head-to-head distance and the highly ordered assembly of the heterocycle molecules might play an important role to afford the two-dimensional proton conducting pathway. As a result, the DOT–40 mol% MDP composite material indicated the high proton conductivity of 4.6 × 10^−4^ S cm^−1^ at 160 °C under the anhydrous conditions.

## 4. Conclusion

We synthesized by the self-assembled nucleobase derivatives, such as 1-dodecylthymine (DOT) and 9-dodecyladenine (DOA), by the reaction with a nucleobase and dodecyl bromide. These derivatives showed a thermal stability by mixing the mono-dodecyl phosphate (MDP). Additionally, the DOT–MDP composite material formed the self-assembled structure with the two-dimensional proton conducting pathway. As a result, the DOT–40 mol% MDP composite material showed the maximum proton conductivity of 4.62 × 10^−4^ S cm^−1^ at 160 °C under anhydrous conditions. In contrast, the proton conduction of the thymine–40 mol% MDP composite material without the self-assembled structure was 1.26 × 10^−6^ S cm^−1^ at 160 °C and this value was two orders of magnitude less than that of DOT–MDP composite material. These results suggested that the two-dimensional proton conducting pathway effectively transfers a proton to the neighbouring site without the vehicular molecule. Therefore, the self-assembled nucleobase derivatives have the potential for use as novel anhydrous proton conductors.

## Conflicts of interest

There are no conflicts to declare.

## Supplementary Material

RA-009-C9RA06841D-s001
