# Supplementary material for: Synthesis of self-assembled nucleobases and their anhydrous proton conductivity
Source: RSC Adv. 2019 Nov 8;9(62):36416–23. doi: 10.1039/c9ra06841d (PMC9074914; doi:10.1039/c9ra06841d)
Supplement: RA-009-C9RA06841D-s001 [file RA-009-C9RA06841D-s001.pdf]

Electronic Supplementary Material (ESI) for RSC Advances.
This journal is © The Royal Society of Chemistry 2019

Supporting Information

Synthesis of self-assembled nucleobase and its anhydrous proton conductivity

Masanori Yamada* and Kento Tanoue

Department of Chemistry, Faculty of Science, Okayama University of Science, Ridaicho, Okayama 700-0005, Japan

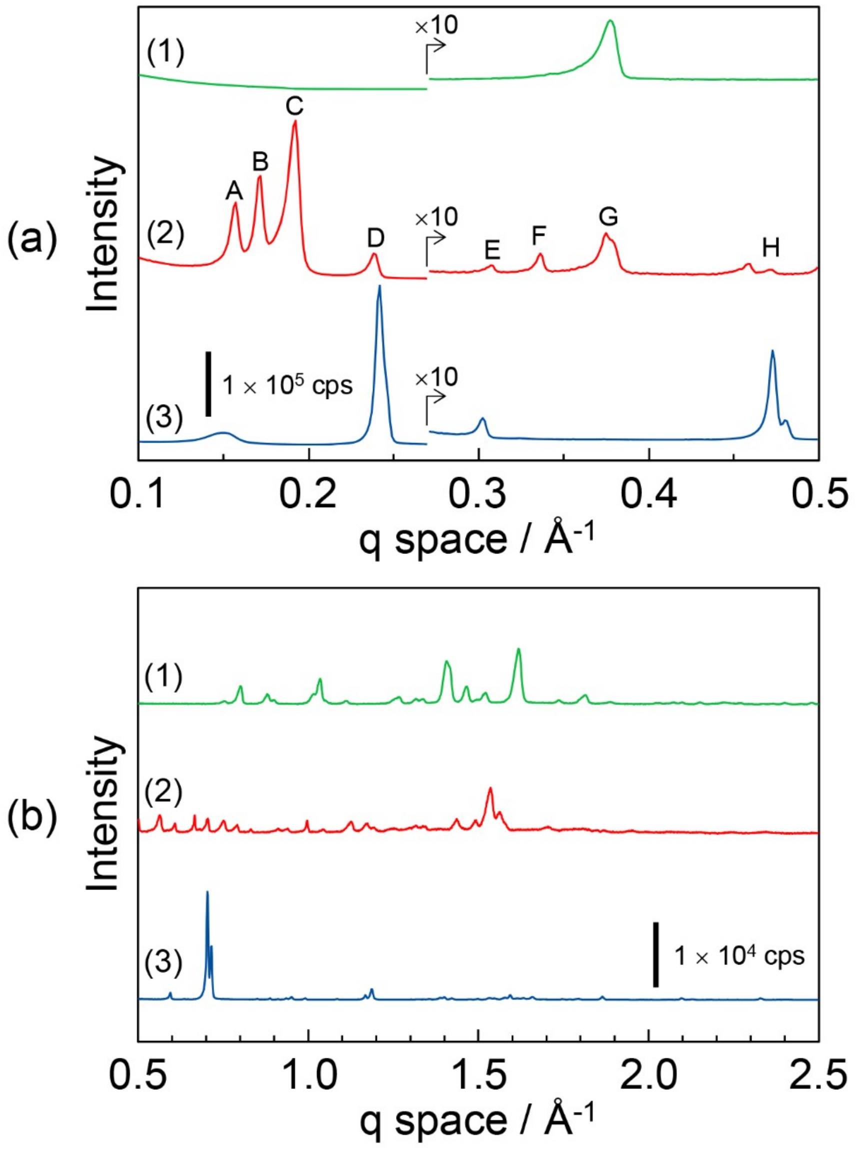

Figure S1. The q space (2π/d-spacing) of (1) DOT material, (2) DOT-40 mol% MDP composite material, and (3) MDP material. (a) and (b) show the q space in the low- and high-angle regions, respectively. The q space was calculated from the d-spacing.

Table S1. The q space of DOT-40 mol% MDP composite material. The q space was calculated from d-spacing. The entry A – H shows the diffraction peaks in Figure S1.

entry	q space / Å^{-1}
A	0.1559
B	0.1704
C	0.1922
D	0.2382
E	0.3083
F	0.3386
G	0.3809
H	0.4751
